# Human versus Artificial Intelligence: Validation of a Deep Learning Model for Retinal Layer and Fluid Segmentation in Optical Coherence Tomography Images from Patients with Age-Related Macular Degeneration

**DOI:** 10.3390/diagnostics14100975

**Published:** 2024-05-08

**Authors:** Mariana Miranda, Joana Santos-Oliveira, Ana Maria Mendonça, Vânia Sousa, Tânia Melo, Ângela Carneiro

**Affiliations:** 1 Department of Surgery and Physiology, Faculty of Medicine of the University of Porto, 4200 Porto, Portugal; 2 Department of Ophthalmology, Centro Hospitalar Universitário of São João, 4200 Porto, Portugal; 3 Electrical and Computer Engineering Department, Faculty of Engineering of the University of Porto, 4200 Porto, Portugal; 4 INESC TEC—Institute for Systems and Computer Engineering, Technology and Science, 4200 Porto, Portugal

**Keywords:** age-related macular degeneration, artificial intelligence, retinal layer segmentation, fluid segmentation, deep learning, machine learning, optical coherence tomography, diagnosis, external validation

## Abstract

Artificial intelligence (AI) models have received considerable attention in recent years for their ability to identify optical coherence tomography (OCT) biomarkers with clinical diagnostic potential and predict disease progression. This study aims to externally validate a deep learning (DL) algorithm by comparing its segmentation of retinal layers and fluid with a gold-standard method for manually adjusting the automatic segmentation of the Heidelberg Spectralis HRA + OCT software Version 6.16.8.0. A total of sixty OCT images of healthy subjects and patients with intermediate and exudative age-related macular degeneration (AMD) were included. A quantitative analysis of the retinal thickness and fluid area was performed, and the discrepancy between these methods was investigated. The results showed a moderate-to-strong correlation between the metrics extracted by both software types, in all the groups, and an overall near-perfect area overlap was observed, except for in the inner segment ellipsoid (ISE) layer. The DL system detected a significant difference in the outer retinal thickness across disease stages and accurately identified fluid in exudative cases. In more diseased eyes, there was significantly more disagreement between these methods. This DL system appears to be a reliable method for accessing important OCT biomarkers in AMD. However, further accuracy testing should be conducted to confirm its validity in real-world settings to ultimately aid ophthalmologists in OCT imaging management and guide timely treatment approaches.

## 1. Introduction

Age-related macular degeneration (AMD) is a leading cause of moderate-to-severe visual impairment (MSVI) [1] and irreversible vision loss in adults aged 50 years and older, in high-income countries [2]. It is estimated to be responsible for 8.7% of the global blindness cases, and, because of population aging, its prevalence is expected to increase [3].

AMD primarily affects the macula, the region of the retina responsible for central vision. This metabolic–inflammatory–vascular disease [4,5,6] is associated with ageing, genetic predisposition [7], and environmental risk factors [8] and is characterised by the deposition of lipid-rich extracellular metabolites within and/or beneath the retinal pigment epithelium (RPE), known as drusen [9]. AMD can be classified into three stages: early, intermediate, and late. Early AMD is defined by the presence of small drusen, while intermediate AMD is associated with medium-sized drusen and retinal pigmentary abnormalities. Late AMD presents in two major forms: geographic atrophy (GA) and neovascular AMD (nAMD) [10]. The latter is caused by macular neovascularisation (MNV), which leads to the accumulation of subretinal fluid (SRF), sub-RPE fluid (sRPEF), and/or intraretinal fluid (IRF).

Optical coherence tomography (OCT) is currently the gold standard for AMD management [11]. Cross-sectional scans of the retina at the micron scale are acquired, where structural features—imaging biomarkers—are identifiable. Central retinal thickness was one of the earliest described; however, others, such as drusen volume, hyperreflective foci quantification [12,13], fluid volume, and pigment epithelial detachment (PED) [14,15], have also been recognised for their insight into disease activity.

Since the rise of artificial intelligence (AI) in medical imaging, retinal OCT has been at the forefront of ophthalmology research [16]. Deep learning (DL) models are currently the state-of-the-art among AI technologies, and they have been shown to be capable for assisting in AMD classification, diagnosis, and prognosis [17,18]; in the ongoing monitoring of the treatment efficacy [19]; and in predicting disease progression [20].

Automated scan analysis using these algorithms is a faster, cost-effective, and fatigue-free process. However, it may have some limitations that affect the quality and accuracy of the results. Therefore, it is crucial to test recently developed and trained methods, such as the BioImagingLab/INESC TEC model [21], on new datasets and externally validate them for their applicability in real-world clinical practice.

This research article contributes to knowledge in the field of optical imaging in diagnosis assisted by AI algorithms, as it presents a novel comparison of the segmentation accuracy to a manually adjusted gold standard. In addition, this article seeks to identify and analyse sites of failure in the DL system, which could represent areas for future improvement of the software.

Thus, the present study aims to evaluate the performance of a DL algorithm in identifying and quantifying retinal layers’ thickness and fluid areas in OCT scans of healthy and AMD eyes. First, quantitatively compare the DL system’s segmentation with the gold standard of the manual adjustment of the Heidelberg Spectralis HRA + OCT automatic segmentation. Subsequently, study whether there is a clear relationship between the thickness layer values extracted and the disease stage, as well as the DL method accuracy in detecting fluid in nAMD. Finally, investigate if the discrepancies between the segmentation methods are related to the disease severity and to the subjective difficulty degree, as perceived by the human eye.

## 2. Materials and Methods

### 2.1. Study Design

A validation study was conducted on normal eyes and eyes with AMD. The study was single-centre, observational, retrospective, and cross-sectional.

The Ethics Committee of the Centro Hospitalar Universitário of São João approved the study protocol for access to and analysis of the patients’ data. Informed consent from patients was not applicable because all the clinical data and OCT images were anonymised by investigators at the Ophthalmology Service. A code number, independent of the participants’ personal data, was generated to protect the patients’ identity. This clinical study was conducted in accordance with the principles outlined in the Declaration of Helsinki.

### 2.2. Setting

OCT scans were collected from healthy eyes and patients diagnosed with intermediate and exudative AMD and who presented for routine clinical care at the Ophthalmology Service of the Centro Hospitalar Universitário of São João, a tertiary referral hospital, between January 2010 and December 2023.

### 2.3. Study Population

A total of 60 fovea-centred cross-sectional OCT B-scans were included from 60 different patients, comprising 20 healthy controls, 20 with intermediate AMD (iAMD), and 20 with exudative AMD (eAMD). Each patient was exclusively assigned to one of the three groups, and only one eye per subject was studied.

A randomised numerical sequence was automatically generated, and each patient was assigned a number at the baseline within their designated group. Inclusion and exclusion criteria were applied until a total of 20 participants were obtained. If the random sequence resulted in the selection of an eye from a patient whose contralateral eye was already included, only one—the one with better visual acuity (whether right or left)—was chosen.

Male and female subjects were evenly distributed, with a 10:10 ratio per group. Controls were required to be 50 years of age or older at the time of the image acquisition, while AMD patients were included if they were 50 years of age or older at the time of the diagnosis. Additionally, an adequate follow-up duration of at least one year after the disease diagnosis was required.

Exclusion criteria for the study included images from patients diagnosed with diabetic retinopathy, high myopia (refractive error ≤ −6.0 dioptres), and/or chorioretinal diseases other than AMD. Patients with OCT scans with poor technical quality due to excessive background noise, imaging artifacts, poor image centration, or reduced visualisation of the retinal layers were also excluded.

**Figure 1:**
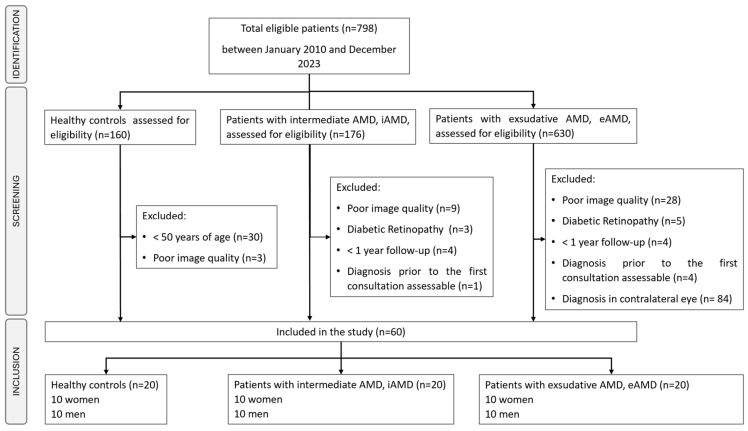
Study flow diagram: patient identification and selection.

In December 2023, 798 participants were assessed for eligibility, including 160 images from healthy controls, 176 scans from individuals diagnosed with iAMD, and 630 OCTs from patients with eAMD. The selection of patients and their allocation to each of the three groups are shown in Figure 1. It is important to note that the retina.pt database contains data from both eyes of each patient. Some cases of unilateral eAMD diagnosis occurred, and the numerically sequenced chosen eye belonged to the contralateral eye, which was either healthy or, more frequently, had intermediate AMD. This scenario was identified in 84 cases, which were also selectively excluded from the third group analysis.

### 2.4. Identification and Image Data Collection

Controls and patients with iAMD were recruited from the electronic health records of the Centro Hospitalar Universitário of São João by applying predefined filters: “Controlos” and “DMI Intermédia”, respectively, which mean “Controls” and “Intermediate AMD”. Participants with eAMD were selected from the online database retina.pt under the category “DMI Avançada (Membrana Neovascular Coroideia)”, which translates to “Late AMD (Choroidal Neovascular Membrane)”.

The OCT images were selected from 6-, 18-, 19-, or 25-line horizontal volume scans acquired using the automatic real-time (ART) function, always centred on the fovea. Enhanced-depth-imaging (EDI) volume scans were not used because of the reduced quality of the retinal layers because this modality typically provides enhanced visualisation of the choroid instead. All the images had a maximum image height of 496 pixels and a variable length, with dimensions of 512 (high-speed mode) or 1024 (high-resolution mode) pixels. No standardisation of the image size was conducted, as these variations do not introduce bias or affect image processing.

The OCT B-scan obtained by the investigators consistently corresponded to the initial image acquired during the first medical consultation. The Heidelberg Spectralis propriety software Version 6.16.8.0 has the capability to conduct retinal segmentation directly within its interface and provides tools to manually adjust each boundary. All the original and segmented images were exported as TIF files. Additionally, images of nAMD were imported into a different platform, the MATLAB system, to complement fluid segmentation. Finally, the original images were imported into the DL software, which performed fully automated segmentation and compared the segmentation results with the gold standard at INESC TEC—Institute for Systems and Computer Engineering, Technology, and Science.

### 2.5. Algorithm Description

Retinal layer segmentations were performed using two distinct methods.

#### 2.5.1. Reference Standard

**Figure 2:**
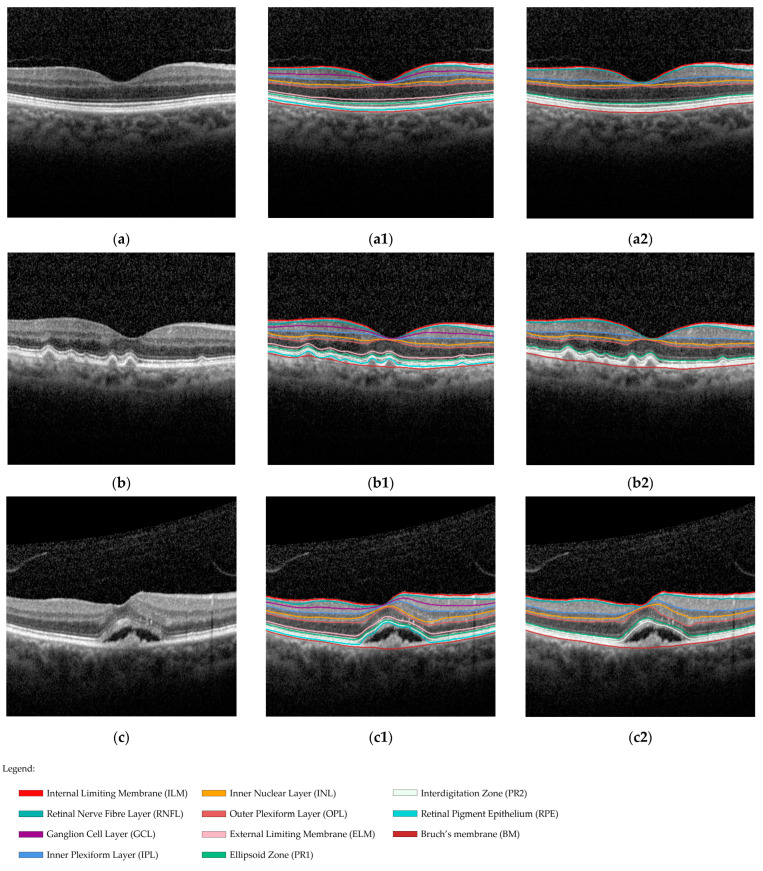
Representative examples of retinal boundary segmentation on OCT B-scan for (**a**) controls, (**b**) iAMD, and (**c**) eAMD patients: (**a1**,**b1**,**c1**) Automated segmentation generated by the Heidelberg System. Retinal layers are as follows from the upper boundary to the lower boundary: internal limiting membrane (ILM), retinal nerve fibre layer (RNFL), ganglion cell layer (GCL), inner plexiform layer (IPL), inner nuclear layer (INL), outer plexiform layer (OPL), external limiting membrane (ELM), ellipsoid zone (PR1), interdigitation zone (PR2), retinal pigment epithelium (RPE), and Bruch’s membrane (BM); (**a2**,**b2**,**c2**) Adjusted automated segmentation, followed by the omission of three layers: ganglion cell layer (GCL), external limiting membrane (ELM), and retinal pigment epithelium (RPE).

The first software that was used was Spectral-Domain Optical Coherence Tomography (SD-OCT, Heidelberg Engineering GmbH, Heidelberg, Germany, Spectralis™ Acquisition Software Version 6.16.8.0) provided with the Heidelberg Spectralis HRA + OCT. This is the proprietary system available to assist ophthalmologists during clinical practice at the Centro Hospitalar Universitário of São João. The software automatically detects ten retinal boundaries. However, during adjusted automated segmentation, three boundaries were omitted from the automated segmentation, as exemplified in Figure 2. This led to the identification of eight final boundaries, thereby enabling a comparative analysis with the segmentation performed by the DL software.

Only for the reference standard (RS) method, fluid segmentation was completed using MATLAB R2023b Update 6 (23.2.0.2485118). The total retinal fluid, including the subretinal fluid (SRF), sub-RPE fluid (sRPEF), and/or intraretinal fluid (IRF), was manually segmented with some automatic suggestions as well.

This first described method was determined as the gold standard for validating the DL model for retinal layers and fluid segmentation.

#### 2.5.2. DL Software

The DL software that was tested was the BioImagingLab/INESC TEC model. The AI algorithm itself identifies the corresponding eight boundaries, starting from the superior limit: internal limiting membrane (ILM), nerve fibre layer/ganglion cell layer (NFL/GLC), inner plexiform layer/inner nuclear layer (IPL/INL), inner nuclear layer/outer plexiform layer (INL/OPL), outer plexiform layer/outer nuclear layer (OPL/ONL), inner segment myoid/inner segment ellipsoid (ISM/ISE), outer segment/retinal pigment epithelium (OS/RPE), and Bruch’s membrane (BM).

**Figure 3:**
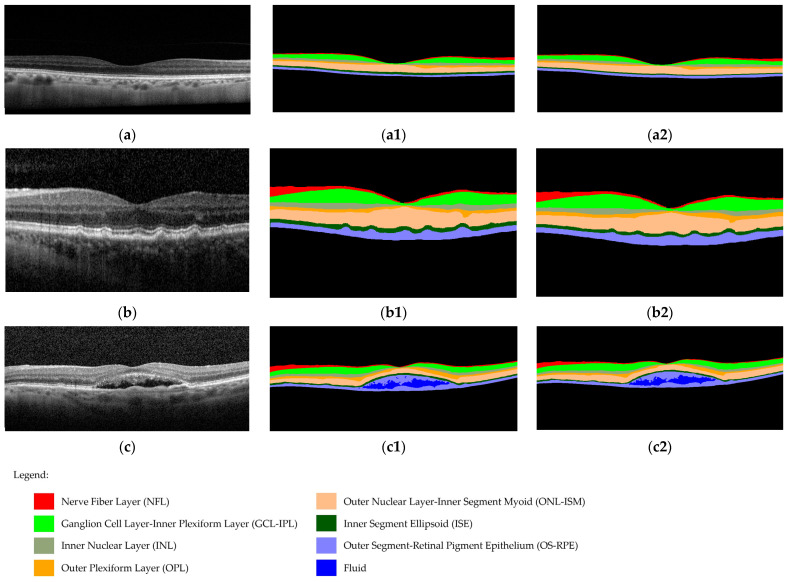
Examples of (**a**) control eye imaging, (**b**) iAMD eye imaging, and (**c**) eAMD eye imaging. Representative examples of retinal layers and fluid segmentation using: (**a1**,**b1**,**c1**) the RS method and (**a2**,**b2**,**c2**) the DL method.

Thus, the seven retinal layers studied in all the groups and the fluid detected in nAMD images are represented in Figure 3.

### 2.6. Image Annotations

The adjusted automated segmentation was carried out by two examiners. Initially, the retinal boundaries were established by a medical student. The annotations automatically generated by the Heidelberg Spectralis software were reviewed and refined through manual adjustments. This involved rearrangement when the system appeared to fail, the annotation was considered to be inaccurate, or, in cases of doubt, the average of both suggestions was represented. The segmentation was then corrected, if necessary, and validated by another observer, an independent medical expert in the field.

During the annotation process, the student assigned a value to each OCT image to reflect the perceived difficulty of the segmentation. This scale was determined subjectively, taking into account the human perception of the image quality in the OCT scan, and was based on a five-level scale: 1 = very easy (no difficulty in establishing any boundary), 2 = easy (difficulty in segmenting a region of the image into one or two boundaries), 3 = medium (difficulty in segmenting a region of the image into more than two boundaries), 4 = difficult (difficulty in segmenting one or two boundaries throughout the entire image), and 5 = very difficult (difficulty in segmenting more than two boundaries throughout the entire image).

The standard fluid segmentation also underwent revision and validation by the same ophthalmologist.

**Figure 4:**
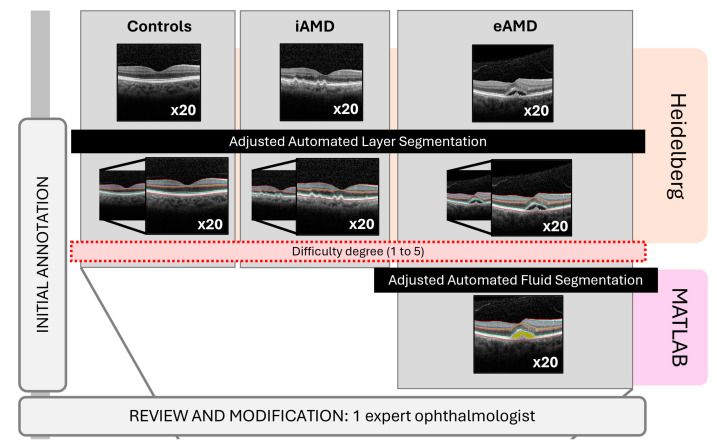

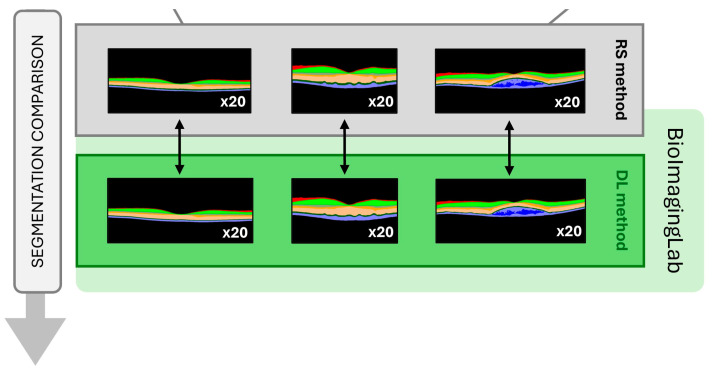
Study flow diagram: image annotations and segmentation comparison. eAMD: exudative AMD; iAMD: intermediate AMD.

Finally, the fully automated segmentation of the retinal layers and fluid was generated by the AI system for the same 60 images. Figure 4 provides an overview of the complete process.

### 2.7. Outcomes

The primary outcomes were the agreement between the DL and RS evaluations and the accuracy of DL software in detecting boundaries and quantifying the thickness of the outer retina (ISE and OS-RPE) and fluid area in nAMD. The secondary outcome was the variance in the disagreement observed between the methods across different image categories: disease stage (objective score) and values of human-perceived difficulty of segmentation (subjective score).

### 2.8. Statistical Analysis

The data were recorded and analysed using Microsoft Excel for Microsoft 365 MSO (Version 2401 Build 16.0.17231.20236) and IBM SPSS Statistics 29.0.0.0 (241). A *p*-value of less than 0.05 was assumed for statistical significance.

All the metrics were measured in pixels. The thickness of the segmented retinal layers was converted to microns (µm) using a vertical scaling factor of 3.87 µm/pixel for all the images. The fluid area was converted to square millimetres (mm^2^) using, additionally, one of two different horizontal scaling factors: 11.35 µm/pixel for images taken in high-speed mode or 5.7 µm/pixel for images captured in high-resolution mode.

Data normality was evaluated through the implementation of the Kolmogorov–Smirnov test and the Shapiro–Wilk test.

To evaluate the performance of the DL system, a paired sample *t*-test was applied to compare the means of the retinal layer thickness and fluid area between the DL and RS methods. This was conducted to determine whether the observed differences were statistically significant. Additionally, the Pearson correlation coefficient was calculated to assess the level of correlation between the variables measured using the two methods. The correlation strength (correlation coefficient value, r) was categorised as weak (0 < r < 0.3 or −0.3 < r < 0), moderate (0.3 ≤ r < 0.7 or −0.7 < r ≤ −0.3), or strong (0.7 ≤ r < 1 or −1 < r ≤ −0.7). Finally, the Dice score, also known as the Dice similarity coefficient, was obtained to evaluate the similarity between the segmentation results and the gold standard. The Dice values range from 0 (no overlap) to 1 (perfect overlap).

To further evaluate the accuracy, the relationship between the thickness of the outer retina extracted by the DL system and the disease stage was investigated through a one-way ANOVA test. Furthermore, a Bland–Altman plot analysis with linear regression was employed to evaluate the concordance between the AI software and RS assessment in quantifying the fluid area.

Finally, the differences in the pairs of measures of the retinal thickness and fluid area between the software types were compared using one-way ANOVA tests. The comparison was made with respect to the degree of disease severity (controls, iAMD, and eAMD), as well as the subjective classification assigned during the RS annotation (very easy, easy, medium, difficult, and very difficult).

## 3. Results

A total of 60 images from 60 patients were included in this study, with 20 images each from healthy controls (mean age ± standard deviation (SD): 63.15 ± 8.00 years, range: 50–79 years), iAMD (mean age ± SD: 71.80 ± 7.72 years, range: 59–83 years), and eAMD (mean age ± SD: 73.80 ± 9.99 years, range: 53–93 years) for a comparative quantitative analysis.

The mean retinal thickness was 307.22 ± 82.99 µm (range: 277.34–332.50 µm) in controls, 319.12 ± 109.79 µm (range: 279.00–395.74 µm) in iAMD, and 332.74 ± 156.91 µm (range: 259.79–384.90 µm) in eAMD.

### 3.1. Performance Evaluation of the DL Software

The assessment of the AI algorithm’s performance involved comparing the automated layer segmentation and the thickness values obtained through the DL system with the measurements obtained from the RS method (Table 1).

The paired sample *t*-test assessment revealed that in the control group, there was no significant difference between the RS evaluation and the segmentation generated by the DL system in the GCL–IPL layer. However, significant differences were observed in all the other layers (*p* < 0.05). In the iAMD group, only the segmentation of the GCL–IPL and ONL–ISM layers showed no significant difference between the two methods, while significant differences were noted in all the other layers (*p* < 0.05). When comparing pairs of metrics for eAMD, the segmentation of all the retinal layers had significant differences, although no significant difference was found in fluid areas between the RS and DL measurements.

All the layers in the control group had strongly positive Pearson correlation coefficients (0.7 ≤ r < 1), except for the ISE and OS–RPE. In the iAMD group, there was a strongly positive (0.7 ≤ r < 1) correlation for the INL and ONL–ISM layers. Similarly, in eAMD, the NFL, ONL–ISM, ISE, and OS–RPE layers and fluid segmentation also showed the same positive correlation range. All the other pairs of layer thicknesses in iAMD and eAMD showed a moderate correlation (0.3 ≤ r < 0.7), except for two layers in each group. Specifically, ISE and OS-RPE in controls, NFL and ISE in iAMD, and GCL–IPL and INL in eAMD had correlation coefficients that were not statistically significant.

Finally, the segmented area showed a near-perfect overlap between the methods in all three groups, as revealed by the Dice coefficient. The mean Dice coefficients for the layers were 0.947 for controls, 0.946 for iAMD, and 0.936 for eAMD. In addition, the fluid had a Dice coefficient of 0.976.

### 3.2. Accessing DL Software’s Accuracy

#### 3.2.1. Retinal Layer Thickness Segmentation

**Figure 5:**
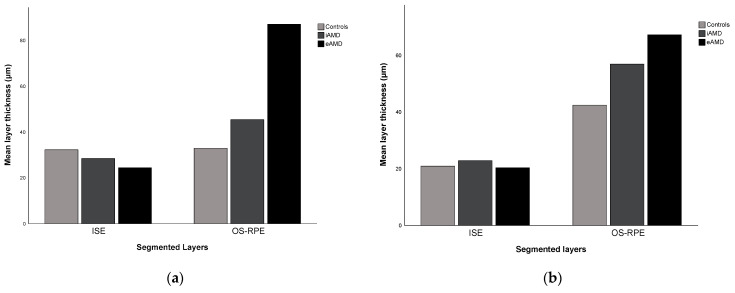
Mean outer retinal thicknesses measured by (**a**) the RS and (**b**) the DL systems across three different disease stages: controls, iAMD, and eAMD. eAMD: exudative AMD; iAMD: intermediate AMD; ISE: inner segment ellipsoid; OS-RPE: outer segment–retinal pigment epithelium.

The one-way ANOVA test showed a statistically significant difference in the mean thickness of the outer retina in the adjusted automated segmentation (*p* < 0.001) and in the AI automatic segmentation (*p* = 0.026) across different disease severities. Figure 5 provides a visual representation of this relationship, particularly in the OS–RPE layer, which showed a mean thickening of 27.06 µm and 12.43 µm with disease, using the RS and the DL methods, respectively.

#### 3.2.2. Fluid Segmentation

**Figure 6:**
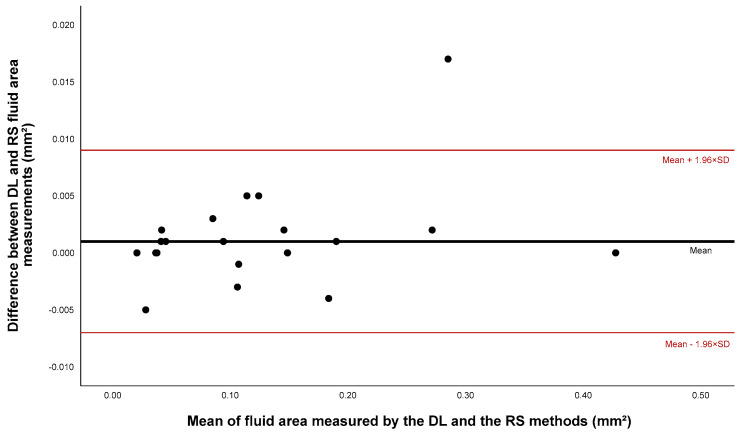
Bland–Altman plot analysis comparing the fluid area measures obtained using the DL software and the RS method. DL: deep learning; RS: reference standard; SD: standard deviation.

To compare the results of the DL system with RS for fluid segmentation, a Bland–Altman plot with differences in the fluid area detected in eAMD eyes is presented in Figure 6. The mean obtained difference was 0.001 ± 0.004 mm^2^. All the measurements, except for one potential outlier, were within the range of ±1.96 SD (0.007–0.009). No apparent trend was observed, suggesting that the DL software tended to either overestimate or underestimate the fluid area, depending on the magnitude of the exudation.

The results of the linear regression analysis did not exhibit any statistical significance (*p* = 0.164), which corroborated the absence of the proportional bias.

### 3.3. Disagreement between Methods in Layers’ Segmentation

**Figure 7:**
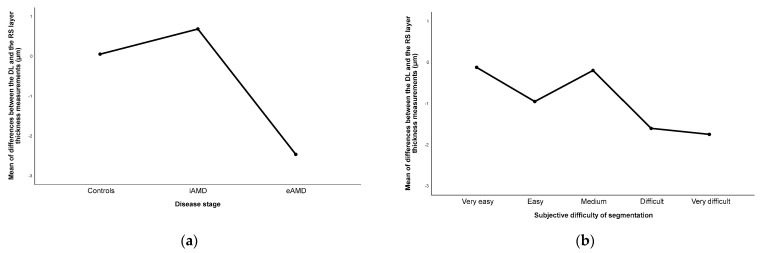
Plots representing the mean differences between the obtained DL and RS layer thickness measurements across (**a**) disease stage and (**b**) subjective difficulty perception of segmentation. DL: deep learning; eAMD: exudative AMD; iAMD: intermediate AMD; RS: reference standard.

A one-way ANOVA test revealed statistically significant differences between both methods for all three stages of the disease severity (*p* < 0.001). However, there was no statistically significant difference across the levels of perceived difficulty in segmentation (*p* = 0.625). Representative graphs illustrating the variance across the objective and subjective scales are found in Figure 7. It was observed that the disagreement increased, as expected, with the disease progression. In iAMD, the DL method gave rise to higher values for the layers’ thickness compared to the RS (positive difference), whereas in eAMD, the DL system resulted in comparatively lower values (negative difference).

## 4. Discussion

This article evaluates the performance of a DL algorithm in the automatic detection and segmentation of retinal layers and fluid in three OCT data groups: healthy controls and two different stages of AMD, intermediate and exudative.

In the absence of an established gold standard, it is a common practice to compare automated AI segmentations with manual annotations, which are typically conducted by independent masked retinal experts, as seen in other published studies [22,23]. However, this approach may not be completely reliable and precise, because of interobserver and intraobserver variability, which introduces some degree of subjectivity [24]. Manual segmentation may also be influenced by limitations in imaging resolution detectable by the human eye. Therefore, even though differences may still exist between human perception and machine interpretation, their merged contribution was considered as being reasonable. Thus, this study innovated by combining human expertise with non-AI automatic suggestions—generated by the Heidelberg Version 6.16.8.0 and the MATLAB Version 23.2.0.2485118 software for the layers and fluid, respectively—to establish a gold standard for comparison with the DL system.

Additionally, this study included real-world OCT images from routine clinical practise rather than images acquired proposedly for investigation purposes. Efforts were made to retain scans of all the levels of complexity, to create balanced groups, ensuring a representative sample to truly test the system.

First, the objective was to determine whether there was agreement in segmentation between the two systems. Significant differences were observed in almost all the pairs of metrics, and in six cases, the Pearson correlation coefficient was not statistically significant. Upon further investigation of the scatter plots between the RS and DL measures, outliers were found in all the cases, except for the ISE layer pairs in controls. Therefore, the non-significant results could be attributed to the presence of outliers in a small sample size. Additionally, in general, there was a moderate-to-strong correlation in all the groups, and the overlap segmentation area of the layers and fluid between both methods was consistently near-perfect. Manual segmentation is a time-consuming and exhaustive task that requires knowledge, a learning curve, and skill. AI-based detection methods could help to overcome the mentioned disadvantages [25]. These results may emphasise the potential this DL method holds in the future as a valuable tool to assist clinics in medical practice, offering a faster, less-fatiguing, and more-systematic approach.

Second, the software accuracy was specifically evaluated by examining the extracted values of the outer retinal thickness. This measurement is expected to be higher in recently diagnosed and treatment-naïve patients. In iAMD, this increase may be caused by basal laminar deposits beneath the RPE, namely, drusen formation, and macula elevation. In eAMD, this thickening may be due to the accumulation of fluid and/or detachment of the RPE. The thinning of some retinal layers may also occur as a consequence of RPE and photoreceptor degeneration, but it is expected to develop over a longer course of the disease [26]. In this study, the outer retina was defined as consisting of the last two measured retinal layers, ISE and OS-RPE, as they were considered as being the most representative layers for this analysis. The results showed a statistically significant difference, particularly because of an increase in the thickness of the OS-RPE layer, as expected. If this biomarker is correlated with the disease stage, it supports this research in confirming its potential for disease classification.

During the evaluation of the software’s capacity to detect and measure the fluid, one outlier was identified. The authors confirmed that it resulted from a vertical shift difference between the fully automatic segmented image (maintaining the original OCT scan position) and the one resulting from the adjusted automated segmentation. This discrepancy likely occurred during the acquisition of the latter image because of a system error, which did not occur in any other case. Despite this outlier, the overall interpretation of results indicates that the DL system’s performance was not affected by the degree of exudation and that it had the capability to accurately detect and quantify the fluid area. Patients with AMD might suffer insidious or sudden painless loss of central or pericentral vision (scotomas) and perception distortion (metamorphopsia) [27], as well as reduced visual acuity under low-luminance conditions and impaired dark adaptation [28]. These symptoms can notably impact daily activities and quality of life [29]. Thus, to ensure timely clinical approaches and treatment decisions, it is essential to distinguish between nonexudative and exudative AMD based on OCT evaluation. In nonexudative AMD, there are currently limited effective therapies for managing atrophy. The disease progression may be slowed with dietary anti-oxidant supplementation [30], and only recently, innovational therapies for atrophic disease have been approved [31]. Therefore, OCT findings consistent with nAMD may be more determinant of the prognosis. If this software tool could aid ophthalmologists in detecting and quantifying retinal fluid, a critical hallmark for initiating anti-vascular endothelial growth factor (anti-VEGF) injections [32,33], which are highly effective, particularly in difficult cases with smaller fluid areas appearing in OCT scans, it could potentially improve patient management.

Finally, a more in-depth investigation was conducted to understand the sources of the discrepancies, exploring both the objective and subjective difficulty levels. This study found an association between a late disease stage and increased disagreement between these methods. However, no statistical difference was found across the values of the human perception of the difficulty in segmentation. The investigation into retinal pathology at different stages of AMD allowed for an understanding of whether the severity of the disease could impact the capacity of the system to accurately recognise and segment retinal layers. This analysis identified areas where the system encountered reasonably higher difficulty, providing insights to guide future technical improvements in the DL algorithm.

Furthermore, the validation of this AI method could enable its application in real-world settings in clinical practice or even serve as an educational tool for new medical professionals, contributing to their learning curve and skill development in interpreting OCT images accurately.

This study had several limitations. First, the enrolled sample size was relatively small, particularly given the single-centre nature of the study. A sample size of 123 OCT images would be required to study a population with an expected prevalence of 8.7%, with a confidence level of 95% and a sample error of 5%. Future research could benefit from a larger population to better assure the generalisability of the findings. Second, the absence of 3D volume measurements for both the retinal layers and fluid because of the need for multiple 2D scans and the segmentation of each image individually. Nevertheless, further investigation should consider seeking volumetric analyses, as previous studies have shown their valuable insights [34,35]. Third, this study was limited to diagnostic evaluation, as longitudinal follow-up data were not collected. Gathering such data could provide a more comprehensive understanding of the predictive capabilities of the developed segmentation system, including its prognostic value [36,37]. Lastly, the study only focused on AMD pathology. It would be important to further include other retinal diseases, such as diabetic retinopathy, central serous chorioretinopathy, epiretinal membrane, and glaucoma, as is conducted in other approaches [38,39,40,41]. Testing the proposed method on a wider range of retinal pathologies would be a valuable challenge and a step towards simulating real-world clinical conditions.

In the future, there is the prospect for training this system in disease classification using biomarkers, such as those studied in this paper, ultimately enabling an autonomous diagnosis that could potentially precede human visual discernment. In a study setting with a considerable sample size, it would be interesting to test the accuracy of the system in classifying diseases and diagnosing them based on the retinal thickness. This could be performed by calculating sensitivities, specificities, and predictive values. Another aspect to explore would be the discriminative capacity of the DL system in distinguishing between the presence and absence of fluid in OCT scans. This could be accomplished by determining a cutoff value and measuring the area under a receiver operating characteristic (ROC) curve (AUC).

In summary, upon the completed validation of this segmentation system, several potential clinical applications may arise. Formal implementation could enable early and more-precise diagnoses, thereby facilitating timely therapeutic interventions, particularly in exudative cases, thus improving patients’ prognoses and quality of life.

## 5. Conclusions

In conclusion, this study contributes to the current knowledge of AI algorithms in OCT imaging. This DL algorithm, when compared to a reference standard, demonstrated moderate-to-strong correlation between metrics, overall high overlap in area segmentation, and the ability to detect outer retinal thickening across disease progression and proved to have high precision for detecting fluid in exudative cases. Moreover, an increased discrepancy between methods was observed in more-advanced disease stages.

Overall, these systems hold the potential to overachieve the precision of the human eye. They could serve as reliable tools that could initially complement existing methods and eventually function autonomously to detect, segment, measure, classify, diagnose, and predict prognoses. Lastly, their integration into patient care and therapeutic assessment could improve clinical outcomes in the ophthalmology field.

## Figures and Tables

**Table 1:** Retinal layer thickness and fluid area obtained from healthy controls, iAMD, and eAMD groups using both RS and DL software. The following layers were considered: ganglion cell layer–inner plexiform layer (GCL–IPL), inner nuclear layer (INL), inner segment ellipsoid (ISE), nerve fibre layer (NFL), outer nuclear layer–inner segment myoid (ONL–ISM), outer plexiform layer (OPL), and outer segment–retinal pigment epithelium (OS–RPE). DL: deep learning; eAMD: exudative AMD; iAMD: intermediate AMD; SD: standard deviation.

	Reference Standard (Mean ± SD)	DL Software (Mean ± SD)	Paired *t*-Test (*p* Value)	Pearson Correlation Coefficient (r)	Dice Coefficient
Healthy Controls (*n* = 20)					
NFL	20.10 ± 10.61	18.23 ± 11.24	<0.001	0.924	0.989
GCL–IPL	71.43 ± 23.32	70.78 ± 24.40	0.101	0.956	0.995
INL	33.00 ± 10.21	32.00 ± 11.38	<0.001	0.980	0.995
OPL	27.47 ± 9.55	26.21 ± 8.88	<0.001	0.971	0.978
ONL–ISM	89.82 ± 20.20	96.83 ± 20.22	<0.001	0.957	0.962
ISE	32.28 ± 5.29	20.91 ± 3.74	<0.001	0.395 ^ⴕ^	0.783
OS–RPE	32.96 ± 3.78	42.43 ± 3.18	<0.001	0.227 ^ⴕ^	0.928
iAMD (*n* = 20)					
NFL	21.96 ± 12.39	19.91 ± 12.61	<0.001	0.382 ^ⴕ^	0.978
GCL–IPL	71.11 ± 23.28	72.03 ± 26.46	0.711	0.496	0.972
INL	33.00 ± 10.07	29.14 ± 11.25	<0.001	0.952	0.951
OPL	27.24 ± 9.37	32.33 ± 11.34	0.002	0.695	0.923
ONL–ISM	89.49 ± 21.93	88.34 ± 22.96	0.274	0.930	0.989
ISE	28.48 ± 6.01	22.84 ± 6.83	<0.001	0.148 ^ⴕ^	0.881
OS–RPE	45.46 ± 21.15	56.91 ± 23.94	<0.001	0.477	0.927
eAMD (*n* = 20)					
NFL	22.08 ± 12.71	19.54 ± 12.69	<0.001	0.814	0.938
GCL–IPL	69.96 ± 25.27	77.76 ± 29.75	0.019	−0.061 ^ⴕ^	0.964
INL	34.64 ± 13.01	30.06 ± 12.44	0.002	0.388 ^ⴕ^	0.938
OPL	31.92 ± 12.33	28.29 ± 15.53	0.003	0.645	0.919
ONL–ISM	71.26 ± 20.54	80.82 ± 24.27	<0.001	0.889	0.922
ISE	24.42 ± 7.61	20.36 ± 18.32	0.003	0.781	0.878
OS–RPE	87.08 ± 62.10	67.28 ± 47.26	<0.001	0.733	0.992
Fluid	0.125 *	0.127 *	0.192	0.999	0.976

* The standard deviation (SD) for the fluid area was not extracted. ^ⴕ^ The results were not statistically significant.

## Data Availability

The data presented in this study are available on request from the corresponding author.
